# Role of afferent and efferent renal nerves in the development of AngII‐salt hypertension in rats

**DOI:** 10.14814/phy2.13602

**Published:** 2018-02-06

**Authors:** Jason D. Foss, Jessica Fiege, Yoji Shimizu, John P. Collister, Tim Mayerhofer, Laurel Wood, John W. Osborn

**Affiliations:** ^1^ Departments of Integrative Biology and Physiology University of Minnesota Minneapolis Minnesota; ^2^ Laboratory Medicine and Pathology University of Minnesota Minneapolis Minnesota; ^3^ Veterinary and Biomedical Sciences University of Minnesota Minneapolis Minnesota

**Keywords:** afferent renal nerves, inflammation, renal denervation, T cells

## Abstract

Hypertension is the leading modifiable risk factor for death worldwide, yet the causes remain unclear and treatment remains suboptimal. Catheter‐based renal denervation (RDNX) is a promising new treatment for resistant hypertension, but the mechanisms underlying its antihypertensive effect remain unclear. We recently found that RDNX attenuates deoxycorticosterone acetate‐salt hypertension and that this is dependent on ablation of afferent renal nerves and is associated with decreased renal inflammation. To determine if this is common to other models of salt‐sensitive hypertension, rats underwent complete RDNX (*n *=* *8), selective ablation of afferent renal nerves (*n *=* *8), or sham denervation (*n *=* *8). Mean arterial pressure (MAP) and heart rate were measure by telemetry and rats were housed in metabolic cages for measurement of sodium and water balance. Rats were then subjected to angiotensin II (AngII)‐salt hypertension (10 ng/kg/min, intravenous + 4% NaCl diet) for 2 weeks. At the end of the study, renal T‐cell infiltration was quantified by flow cytometry. AngII resulted in an increase in MAP of ~50 mmHg in all three groups with no between group differences, and a transient bradycardia that was blunted by selective ablation of afferent renal nerves. Sodium and water balance were unaffected by AngII‐salt treatment and similar between groups. Lastly, AngII infusion was not associated with T‐cell infiltration into the kidneys, and T‐cell counts were unaffected by the denervation procedures. These results suggest that AngII‐salt hypertension in the rat is not associated with renal inflammation and that neither afferent nor efferent renal nerves contribute to this model.

## Introduction

High blood pressure (hypertension) is estimated to affect over one billion people and, due to its association with stroke, heart failure, and kidney disease, is now the leading risk factor for death worldwide (Kearney et al. 2005; Murray and Lopez 2013). Despite this, an unacceptably high percentage of patients fail to achieve healthy blood pressure with current treatments (Judd and Calhoun 2014; Yoon et al. 2015). This is in part due to a failure to fully understand the mechanisms involved in the development and maintenance of hypertension. The sympathetic nervous system is one known contributor to the pathogenesis of hypertension (Guyenet 2006; Grassi et al. 2015; Joyner and Limberg 2016). More specifically, the renal nerves play an important role in various models of experimental hypertension, and recent clinical trials have shown that ablation of renal nerves (renal denervation; RDNX) lowers arterial pressure in some drug‐resistant patients (Osborn and Foss 2017). Although the failure of some clinical trials to demonstrate efficacy has raised questions regarding the viability of this therapy (Gulati et al. 2016), the early results of the SPYRAL HTN‐OFF MED trial, in which an improved catheter and trial design were used, indicate RDNX is effective in hypertensive patients (Townsend et al. 2017).

It is clear that further preclinical research is needed to translate RDNX from preclinical research to the clinic. Specifically, the mechanisms underlying the antihypertensive effect of RDNX are not known. One important point that remains to be clarified is whether the effects of RDNX are due to ablation of afferent (sensory) or efferent (sympathetic) renal nerves (DiBona and Esler 2010). Ablation of sympathoexcitatory afferent renal nerves could, in theory, decrease arterial pressure secondary to reductions in sympathetic drive to the kidney and other organs. Alternatively, ablation of efferent renal nerves could, in theory, reduce renal vascular resistance, renin release, and renal sodium and water reabsorption (Osborn and Foss 2017). While both hypotheses are logical, a definitive answer remains elusive.

Moreover, because the causes of hypertension are many, it is unlikely that the efficacy of one form of treatment will be universal and, therefore, the mechanisms underlying the treatment effect will vary from patient to patient. To this point, we have recently shown that RDNX attenuates hypertension in both the deoxycorticosterone acetate (DOCA)‐salt and Dahl Salt‐Sensitive (DS) rat models (Banek et al. 2006; Foss et al. 2016); however, whereas this response was entirely due ablation of *afferent* renal nerves in the DOCA‐salt rat, the opposite was true in the DS rat since selective afferent renal nerve ablation had no effect on this model of salt‐sensitive hypertension (Banek et al. 2006; Foss et al. 2016). These findings speak to the complexity of RDNX as an effective treatment for hypertension and highlight the importance of a mechanistic understanding of the therapeutic effect of RDNX, and identifying the conditions under which RDNX may be effective.

One such condition may be renal inflammation. The immune system has recently been shown to contribute to the pathogenesis of hypertension. Specifically, inflammation of the vasculature, brain and kidneys contributes to chronic increases in arterial pressure (Zubcevic et al. 2014; De Miguel et al. 2015; McMaster et al. 2015). Moreover, some studies suggest that renal inflammation specifically may be directly caused by increased renal nerve activity. For example, Xiao et al. (2015) demonstrated that angiotensin II (AngII)‐induced hypertension in mice is attenuated by RDNX and that this is accompanied by reduced renal inflammation in a pressure‐independent manner. Since specific ablation of afferent renal nerves had no effect on the pathogenesis of hypertension in that study, it was concluded that the antihypertensive effect of RDNX was due to ablation of *efferent* renal nerve mediated renal inflammation (Xiao et al. 2015). Similar to Xiao and colleagues studying the murine AngII model (Xiao et al. 2015), we recently reported that RDNX attenuates hypertension and renal inflammation in the rat DOCA‐salt model (Banek et al. 2006). However, we also reported that resting afferent nerve discharge is elevated in DOCA‐salt rats and that this is correlated with a marked increase in a number of inflammatory cytokines in the kidney. In contrast to the study of Xiao and colleagues (Xiao et al. 2015), we observed that afferent‐specific renal nerve ablation attenuated hypertension in this model to the same degree as total RDNX (Banek et al. 2006). These findings led to the conclusion that, although renal inflammation may be dependent on *efferent* renal nerves, hypertension was driven by increased *afferent* renal nerve activity, possibly secondary to renal inflammation (Banek et al. 2006).

This study was motivated by two factors. First, in light of our recent reports in which the role of afferent and efferent nerves in mediating the antihypertensive response to RDNX was opposite in two rat models of salt‐sensitive hypertension (Banek et al. 2006; Foss et al. 2016); we felt it was important to explore this question in another salt‐dependent model. Second, evidence for an interaction between the autonomic and immune systems in the pathogenesis of hypertension is largely based on studies in the mouse using the AngII‐induced model (Lob et al. 2010; Marvar et al. 2010; Xiao et al. 2015). Since we recently reported an important interaction between renal nerves, renal inflammation, and hypertension in DOCA‐salt rats (Banek et al. 2006), we felt it was important to investigate whether the same relationship exists in the AngII‐salt rat model. To do this, we compared the effects of total (efferent + afferent) and afferent‐specific renal nerve ablation to sham treatment on arterial pressure, renal inflammation, and body fluid homeostasis in rats administered AngII in combination with a high salt diet.

## Materials and Methods

### Animals and general procedures

A total of 29 male Sprague–Dawley rats were purchased from Charles River Laboratories (Wilmington, MA) and housed in pairs in a temperature and light controlled room until the beginning of the study. Rats were allowed access to standard rat chow and distilled water ad libitum during this pre‐experimental period. All procedures were approved by the University of Minnesota Animal Care and Use Committee and were conducted in accordance with the institutional and National Institutes of Health guidelines. For all surgeries, rats were anesthetized with 2.0% isoflurane (Phoenix Pharmaceutical, St. Joseph, MO). Atropine sulfate (0.4 mg/kg, intraperitoneal; West‐Ward Pharmaceuticals, Eatontown, NJ), gentamicin sulfate (10 mg/kg, intramuscular; Hospira, Lake Forest, IL) and ketoprofen (5 mg/kg, subcutaneous; Fort Dodge Animal Health, Overland Park, KS) were administered prior to surgery. For 3 days following surgery, rats received daily intravenous injections of 15 mg ampicillin (Pfizer, New York, NY).

### Denervation procedures

#### Total RDNX

Total denervation (afferent + efferent) of the kidneys was performed as previously described (Foss et al. 2015). Briefly, renal nerves were exposed through a midline abdominal incision. All visible renal nerve fibers were sectioned and the renal artery and vein were brushed with a small piece of gauze soaked in 10% phenol in ethanol. The area was then dried and the procedure repeated on the contralateral side.

#### Selective ablation of afferent renal nerves (renal‐CAP)

Selective ablation of afferent renal nerves was performed as previously described (Esler and Guo 2016). Briefly, a midline abdominal incision was made and the left renal artery and vein were exposed through a small hole in the peritoneal membrane. The fat surrounding the renal vessels was then dissected away and parafilm was placed underneath. A small piece of gauze soaked in a capsaicin solution (33 mmol/L in 5% ethanol, 5% Tween 80 and 90% normal saline) was wrapped around the vessels for 15 min. The gauze and parafilm were removed, the area was dried, and the procedure was repeated on the contralateral side.

#### Sham surgery

The left and right renal nerves were visualized through a midline abdominal incision without physical disruption of the area.

### Experimental protocol

Male Sprague–Dawley rats (275–350 g) were placed on a high salt diet (4% NaCl; Research Diets, New Brunswick, NJ) and instrumented with radio telemeters (model TA11PA‐C40, DSI; Intl. St. Paul, MN) for monitoring of mean arterial pressure (MAP) and heart rate (HR) as previously described (Veitenheimer and Osborn 2011). Rats were also subjected to either SHAM (*n *=* *8), RDNX (*n *=* *8) or renal‐CAP (*n *=* *8) surgery as described above. In addition, a venous catheter was placed in the left femoral vein for infusion of saline or AngII. The catheter was tunneled subcutaneously, exited between the scapulae, passed through a rubber harness and a protective flexible spring and was attached to a freely rotating single‐channel hydraulic swivel.

Rats were then individually housed in metabolic cages (Nalge Nunc, Rochester, NY) and allowed to recover for 7 days during which time a continuous intravenous infusion of sterile 0.9% NaCl solution (7 mL/day) was maintained. Three days of baseline data were then collected (see below for details) and then rats were infused with AngII (10 ng/kg/min, intravenous) for the remainder of the protocol.

At the end of the study, rats were anesthetized with isoflurane, euthanized by exsanguination and kidneys were removed immediately after death. The renal artery, renal vein, ureter and capsule were removed and the kidneys were placed in a cold normal saline bath for further dissection. Kidneys were halved and one half was further processed for flow cytometry (see below). From the other half, renal parenchymal samples were taken from the poles and lateral portion of the kidney and flash frozen in liquid nitrogen. The renal pelvis was then carefully dissected from the remaining portion of kidney and flash frozen in liquid nitrogen. All frozen samples were stored at −80°C until being assayed.

The parenchymal samples were assayed for norepinephrine (NE) using HPLC as previously described (Li et al. 2010). The isolated renal pelvic samples were assayed for calcitonin gene‐related peptide (CGRP) using a commercially available ELISA kit (Item Number 589001; Cayman Chemicals; Ann Arbor, MI). Tissues were homogenized in 1M acetic acid and CGRP was extracted using C18 Sep‐columns (Item Number Y‐1000; Peninsula Laboratories; San Carlos, CA). To eliminate any interassay variance, all pelvic samples were run on a single 96 well ELISA plate.

### Daily measurements

The transmitter signal was monitored by a receiver (model RPC‐1; Data Sciences, St. Paul, MN, USA) mounted on the back of the metabolic cage and connected to a Data Exchange Matrix (Data Sciences, Int). The arterial pressure signal was sampled at 500 samples/sec for 10 sec every 4 min using commercially available software (Data Sciences, Int.). HR was determined from the arterial pressure profile using the same software. 24‐h averages of MAP and HR were determined and plotted for each day of the study.

Twenty‐four‐hour food intake, water intake and urine output were measured gravimetrically. Twenty‐four‐hour sodium intake was calculated by multiplying food intake (g) and sodium content of the diet (4.0% NaCl = 0.6844 mmol Na^+^/g food). 24 h sodium excretion was calculated by multiplying 24 h urine output (mL) and urinary sodium concentration (mmol Na^+^/mL), which was measured using an ion‐specific electrode (NOVA‐5+ electrolyte analyzer; Nova Biomedical, Waltham, MA). Twenty‐four hour sodium and water balances were calculated as 24 h intake minus 24 h excretion.

### Flow cytometry

Kidneys were cut into 10 mm pieces and were incubated in RPMI 1640/5%FCS/2 mmol/L MgCl2/2 mmol/L CaCl2 with 100 U/mL collagenase I (#4197; Worthington, Lakewood, NJ, USA) for 45 min/37°C. Lymphocytes from kidney were purified on a 44/67% Percoll gradient (800*g* at 20°C for 20 min). Single‐cell suspensions were stained with PE anti‐CD3 (1F4), PE‐Cy7 anti‐CD4 (OX‐35), V450 anti‐CD8*α* (OX‐8), Biotin anti‐CD80 (3H5), Biotin anti‐CD86 (24F) (BD Pharmingen, San Jose, CA, USA), and FITC anti‐CD44 (OX‐49), AF647 anti‐CD62L (OX‐85) (Biolegend, San Diego, CA, USA), and followed with APCef780‐SA (eBioscience) in 1XHBSS/2%CS. For identification of Treg, FITC anti‐CD25 (OX‐39) (Biolegend, San Diego, CA, USA) and intracellular staining for APC anti‐FoxP3 (FJK‐16s) was performed using the FoxP3 kit in accordance with the manufacturer's directions (eBioscience). Samples were acquired on a LSRFortessa flow cytometer (BD Pharmingen) and analyzed using FlowJo software (TreeStar).

Due to processing issues, not all kidneys from SHAM, RDNX, and renal‐CAP rats were analyzed by flow cytometry. An additional five male Sprague–Dawley rats were euthanized and kidneys were harvested in the same way as experimental rats to determine if AngII‐salt hypertension was associated with increased renal T‐cell infiltration compared to naïve rats.

### Statistical analysis

MAP, HR, and sodium and water balance data were analyzed by two‐way analysis of variance (ANOVA) for repeated measures followed by the Bonferroni method for post hoc comparisons using GraphPad Prism v6 (GraphPad Software, San Diego, CA). Renal T‐cell counts, CGRP and NE data were analyzed by one‐way ANOVA. A *P* value less than 0.05 was considered to be statistically significant.

## Results

### Effect of RDNX and renal‐CAP on renal content of neurotransmitters

**Figure 1:**
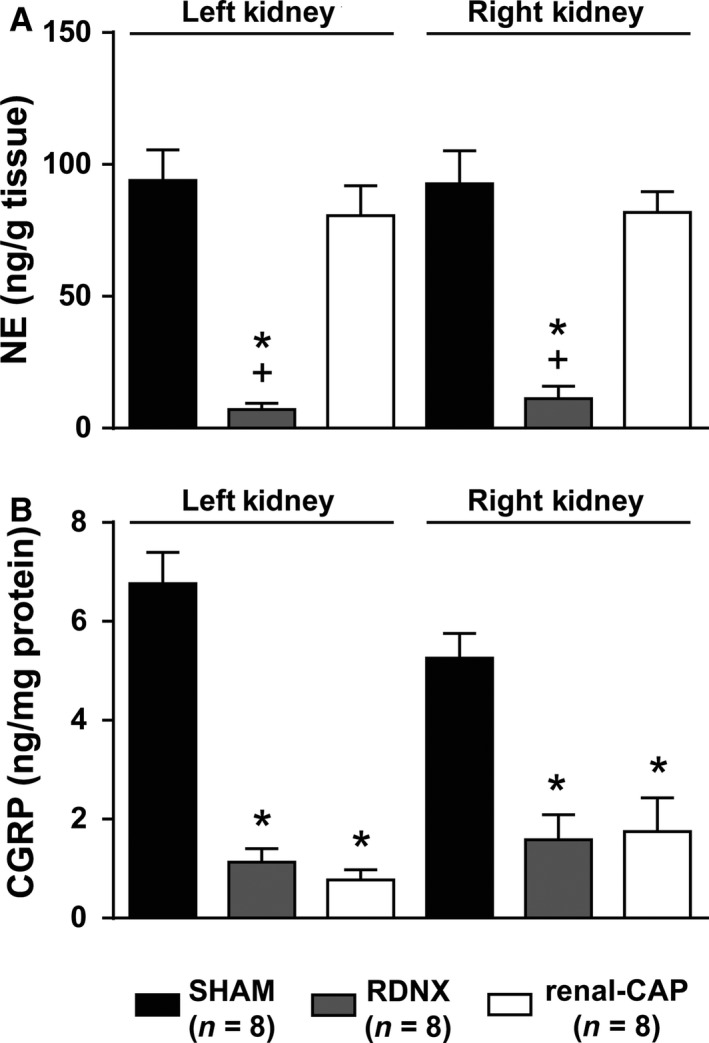
Content of (A) efferent nerve marker, norepinephrine (NE) and (B) afferent nerve marker, calcitonin gene‐related peptide (CGRP). *P <* *0.05 versus SAHM, ^+^ *P *<* *0.05 versus renal‐CAP.

To confirm the ablation of efferent and afferent renal nerves, kidneys were assayed for the efferent nerve marker, NE and the afferent nerve marker, CGRP. RDNX drastically decreased renal NE (Fig. 1A) and CGRP (Fig. 1B) content in both the right and left kidneys, thus confirming complete denervation of both kidneys. Renal‐CAP resulted in a similar decrease in renal CGRP content as RDNX (Fig. 1B), but had no effect on renal NE content (Fig. 1A), thus confirming selective ablation of afferent and not efferent renal nerves in both kidneys.

### Effect of RDNX and renal‐CAP on MAP, HR, and sodium/water balance

**Figure 2:**
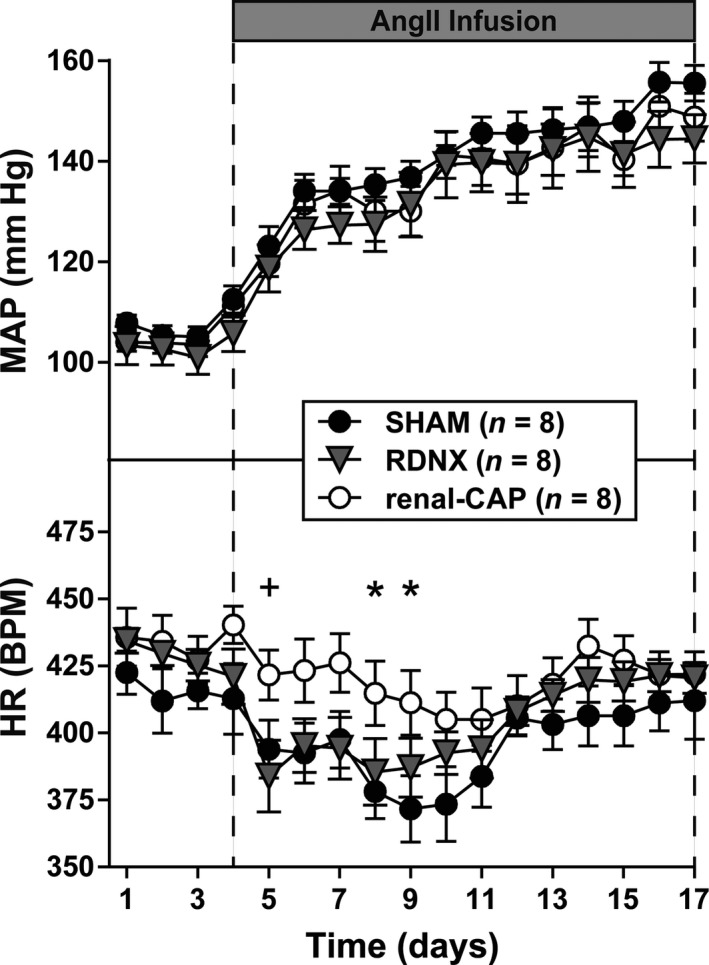
Mean arterial pressure (MAP) and heart rate (HR). There were no differences in MAP between groups. *P <* *0.05 renal‐CAP versus SAHM, ^+^ *P *<* *0.05 renal‐CAP versus RDNX. RDNX, renal denervation

To assess whether afferent or efferent renal nerves contribute to the development of AngII‐salt hypertension, SHAM, RDNX, and renal‐CAP rats were implanted with a radiotelemeters and an intravenous catheter, fed a high salt diet and infused with AngII for 2 weeks. As shown in Figure 2, MAP was ~100 mmHg in all groups at baseline. Over 2 weeks of AngII infusion, MAP increased to ~150 mmHg in all groups. There were no statistically significant differences in MAP between groups at any time point.

HR was also similar between groups at baseline. Upon AngII infusion, HR transiently decreased in all groups and then returned to baseline levels; however, this transient bradycardic response to AngII was blunted somewhat in renal‐CAP rats.

**Figure 3:**
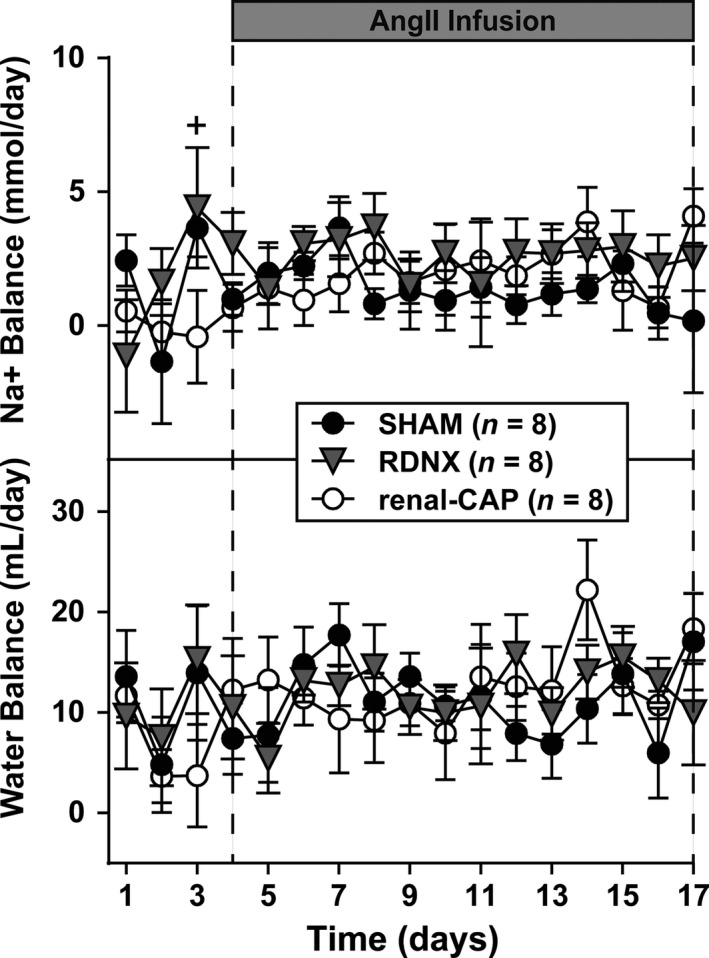
24 h sodium (Na+) and water balance. There were no differences in water balance between groups. ^+^ *P* < 0.05 renal‐CAP versus RDNX. RDNX, renal denervation

Because renal nerve activity is known to modulate renal excretory function, daily sodium, and water balance measurements were made. With the exception of the third day of baseline, 24 h sodium, and water balance measurements were similar between groups throughout the protocol (Fig. 3). Moreover, sodium and water balance were unchanged following AngII infusion.

### Effect of RDNX and renal‐CAP on recruitment of T cells to the kidneys in AngII‐salt hypertension

**Figure 4:**
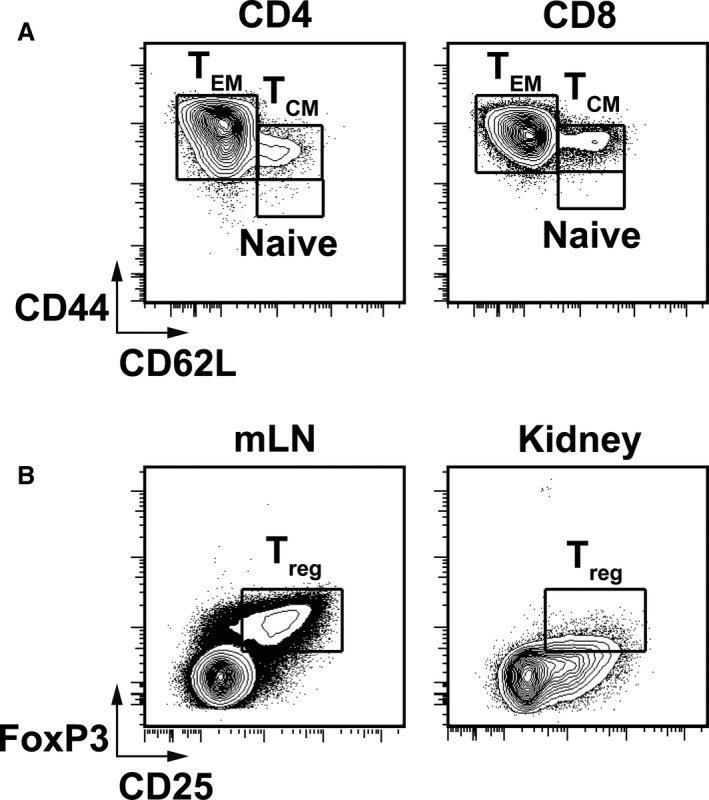
Sample flow cytometry gating. A. Sample gating for CD4+ and CD8+ Naïve T cells, effector memory T cells (TEM) and central memory T cells (TCM). B. Sample gating for CD4+ CD8‐ regulatory T cells (Treg) in mesenteric lymph node (mLN) and kidney.

**Figure 5:**
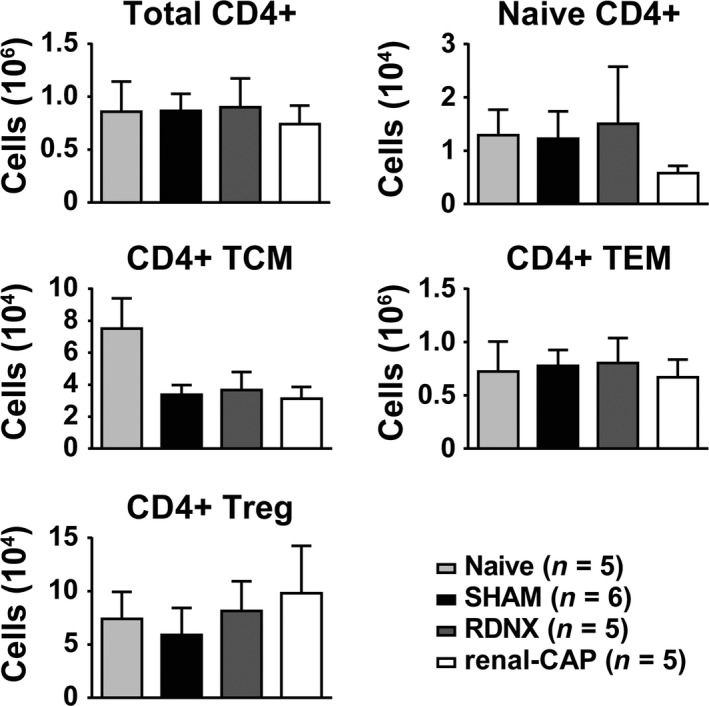
Number of CD4+ T cells per kidney as quantified by flow cytometry. There were no differences between groups in total CD4+ cells, naïve CD4+, CD4+ central memory T cells (TCM), CD4+ effector memory cells (TEM) or CD4+ T regulatory cells (Treg).

**Figure 6:**
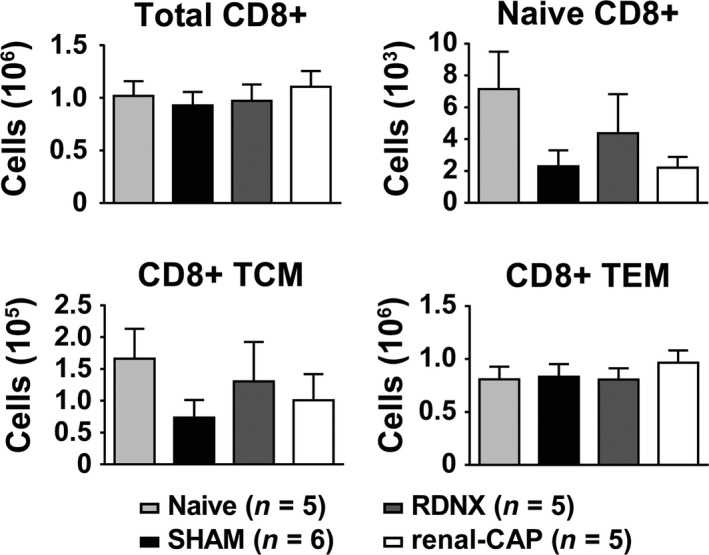
Number of CD+ T cells per kidney as quantified by flow cytometry. There were no differences in total CD8+ cells, naïve CD8+ T cells, CD8+ central memory T cells (TCM) or CD8+ effector memory cells (TEM).

Recent reports have implicated renal nerves in the development of hypertension through recruitment of T cells into the kidneys (Xiao et al. 2015). Therefore, infiltration of T cells into the kidneys was assessed by flow cytometry using the gating strategy shown in Figure 4. Compared to naïve rats, AngII‐salt hypertension did not significantly increase the number of CD4+ T cells in the kidney (Fig. 5). This was true for total CD4+ cells as well as all subsets of CD4+ cells assessed including naïve CD4+, CD4+ central memory T cells (TCM), CD4+ effector memory cells (TEM) and CD4+ T regulatory cells (Treg). Moreover, neither RDNX nor renal‐CAP affected renal CD4+ T‐cell counts compared to SHAM. Likewise, AngII did not increase infiltration of CD8+ T cells into the kidneys and there were no differences in renal CD8+ T cells between groups (Fig. 6). This was true for total CD8+ cells as well as naïve CD8+ T cells, CD8+ TCM, and CD8+ TEM cells.

## Discussion

Recent clinical trials of catheter‐based RDNX have yielded promising, but sometimes conflicting results. While many studies have shown a positive antihypertensive effect of the procedure, other trials have failed to show any benefit (Davis et al. 2013; Gulati et al. 2016). Although it is not entirely clear why some trials have failed to demonstrate efficacy, Esler and Guo (2016) recently argued that the use of an intravascular catheter‐based RDNX method for the treatment of hypertension has sound physiological basis and the failure of recent clinical trials is likely due to factors such as poor experimental design and inadequate denervations. Indeed, the inability to confirm the extent of RDNX in humans is a major limitation of this method. The positive results of the SPYRAL HTN‐OFF MED trial in which an improved catheter and trial design were used indicate that RDNX is indeed effective in hypertensive patients (Townsend et al. 2017). Taken together, these results highlight the need for more in depth preclinical research to determine the conditions under which RDNX lowers arterial pressure and the mechanisms of this antihypertensive therapy.

Animal studies are particularly useful in this regard as the hypertensive stimuli are tightly controlled and complete denervation of the kidneys is consistently achieved via extravascular surgical approaches, which can be confirmed by post‐mortem measurement of renal neurotransmitter content. Moreover, animal studies allow for high‐quality quantitative assessment of parameters such as 24 h ambulatory arterial pressure, sodium and water balance, and renal inflammation. Lastly, animal studies allow for the exploration of the basic physiological mechanisms by which RDNX decreases arterial pressure in various hypertensive models, which is critical to the development of neuromodulatory therapies targeting renal nerves.

### Animal model variability in the contribution of renal nerves to hypertension

Our laboratory and others have studied the role of renal nerves in numerous rat models of hypertension (Osborn and Foss 2017). Much like with human hypertension, RDNX effectively lowers arterial pressure in some models, and not in others (Osborn and Foss 2017). Moreover, we recently developed a method to specifically target afferent renal nerves for ablation that has allowed us to assess the contribution of afferent and efferent renal nerves to the pathogenesis of hypertension (Foss et al. 2015). Using this new method of afferent renal nerve ablation, we have found that there are differences between animal models. For example, selective ablation of afferent renal nerves by periaxonal capsaicin treatment attenuates DOCA‐salt hypertension in a manner identical to total RDNX (afferent + efferent) (Banek et al. 2006; Foss et al. 2015). This suggests that the antihypertensive effect of RDNX in this model is due to ablation of afferent renal nerves. In contrast, we found that in another rat model of salt‐sensitive hypertension, the DS rat, RDNX decreases arterial pressure but the selective ablation of afferent renal nerves has no effect (Foss et al. 2016). This study suggests that the antihypertensive response to RDNX is entirely due to ablation of efferent nerves. It is important to recognize that these studies were conducted in the same laboratory, using the same methods for renal nerve ablation, measurement of arterial pressure, and confirmation of RDNX. As such, these studies suggest that, although RDNX decreases arterial pressure in both models, this response is due to ablation of afferent renal nerves in one model (DOCA‐salt) and efferent nerves in the other (DS).

In this study, we applied the same approach to investigate the contribution of afferent and efferent nerves to a third model of salt‐sensitive hypertension – the AngII‐salt rat. We observed that, in contrast to the DOCA‐salt and DS models, total RDNX had no effect on the pathogenesis of hypertension in AngII‐salt rats. In this study, AngII was infused intravenously at a rate of 10 ng/kg/min in rats consuming a 4% NaCl diet. It should be noted that our group has also studied a slightly different model of AngII‐salt hypertension in which AngII is administered subcutaneously at a rate of 150 ng/kg/min to rats consuming a 2% NaCl diet. In this model RDNX also had no effect on arterial pressure (King et al. 2007) consistent with our finding that renal nerve activity is actually decreased during AngII‐salt administration (Yoshimoto et al. 2010). In combination with this study, these findings strongly suggest that renal nerves do not contribute to the development of AngII‐salt hypertension regardless of the route of AngII administration (subcutaneously or intravenous) or whether rats are consuming 2% or 4% NaCl diet.

It is important to note that we have observed variability within our own group regarding the effect of RDNX on AngII‐salt hypertension in that we previously reported RDNX attenuates the development of AngII‐salt hypertension using the same model as this study (10 ng/kg/min i.v. AngII plus 4% NaCl diet) (Hendel and Collister 2006). What is the explanation for this variability? The most likely explanation is that, for unknown reasons, the hypertensive response to AngII + salt in SHAM rats in this study was ~20 mmHg less than our earlier report (Hendel and Collister 2006). We hypothesize that renal nerves contribute to AngII‐salt hypertension once a certain threshold of arterial pressure is reached due to changes in the blood–brain barrier in AngII‐induced hypertension as recently reported (Biancardi et al. 2014). We propose that in our previous study, the more robust response to AngII+ salt causes a breakdown of the blood–brain barrier, allowing AngII to access noncircumventricular organ sympathoexcitatory centers in the brain. This is consistent with the report that RDNX attenuates hypertension induced by chronic intracerebroventricular infusion of AngII in rats consuming a high salt diet (Osborn and Camara 1997). However, additional studies are needed to test this hypothesis.

In addition to the mixed results regarding the effect of RNDX on rat models of AngII‐salt hypertension, RDNX attenuates the development of AngII‐induced hypertension in the mouse (Xiao et al. 2015). Moreover, this response is due to ablation of efferent renal nerves, since selective ablation of afferent renal nerves by periaxonal capsaicin has no effect on arterial pressure (Xiao et al. 2015). The mechanism by which RDNX decreases arterial pressure in the AngII‐induced mouse model may be related to trafficking of T cells into the kidney (Xiao et al. 2015) as discussed further below. It remains unclear at the present time why RDNX decreases arterial pressure in the AngII mouse but not the AngII‐salt rat model. This may be due to species differences or the contribution of a high salt to AngII‐induced hypertension. This further underscores the idea that RDNX may not be an effective antihypertensive therapy for all forms of hypertension and that, when it is, the mechanism underlying the effect may differ as well.

Finally, it is interesting to note that while there were no differences in MAP between groups, selective ablation of afferent renal nerves blunted the bradycardic response to AngII observed in SHAM and RDNX rats. While it remains unclear why this would be, we recently found a similar effect in high‐salt fed rats (Foss et al. 2015). Further experiments are needed to clarify the role of afferent renal nerves in long‐term regulation of heart rate.

### Effect of RDNX on sodium and water balance

Sympathetic innervation of the kidneys alters renal excretory function, at least acutely. To determine whether afferent or efferent renal nerves are important in long‐term regulation of sodium and water homeostasis, we measured sodium and water intake and excretion and calculated 24 h sodium and water balances. Despite extensive denervation of both kidneys as confirmed by renal NE content, neither selective afferent RNDX nor total RDNX affected sodium or water balance as compared to Sham control rats. This in agreement with previous studies in Sprague–Dawley (Jacob et al. 2003) and DS (Foss et al. 2013, 2016) rats. We have found in two separate studies that RDNX decreases arterial pressure in hypertensive DS rats in the absence of changes in cumulative sodium and water balance (Foss et al. 2013, 2016). In addition, we have reported that RDNX has no effect on sodium and water balance in Sprague–Dawley rats fed either high‐ or low‐salt diets (Jacob et al. 2003). Collectively, these results suggest that other regulatory systems compensate for the loss of renal sympathetic innervation in order to maintain long‐term sodium and water balance in these models.

### Renal nerves, renal inflammation, and hypertension: Is there a link?

It is becoming increasingly clear that inflammation of the kidneys and vasculature contributes to the pathogenesis of hypertension (McMaster et al. 2015). It has recently been hypothesized that the sympathetic nervous system plays an important role in this hypertensive inflammatory process and that renal nerves directly contribute to renal inflammation (Lob et al. 2010; Marvar et al. 2010). This concept is supported by reports that RDNX reduces renal inflammation in DOCA‐salt‐treated rats (Banek et al. 2006) and AngII‐infused mice, (Xiao et al. 2015). In addition, we found a direct correlation between renal inflammation and resting afferent renal nerve activity in DOCA‐salt rats and that afferent‐specific renal nerve ablation attenuates hypertension in this model (Banek et al. 2006). We hypothesized that, in the DOCA‐salt rat, renal inflammation is driven in part by efferent renal nerves, and that this renal inflammation then drives afferent renal nerve activity and hypertension (Banek et al. 2006).

In contrast, using the identical methods for afferent and total renal nerve ablation, measurement of arterial pressure, and flow cytometry used to study the DOCA‐salt model (Banek et al. 2006), this study found no effect of RDNX on the pathogenesis of hypertension or renal inflammation in the AngII‐salt rat. In fact, in contrast to what has been reported in several studies in mouse model of AngII‐induced hypertension (Kirabo et al. 2014; Saleh et al. 2015; Xiao et al. 2015), AngII‐salt hypertension in the rat was not associated with increased T‐cell counts in the kidneys compared to naïve controls. The reasons for such disparate responses regarding infiltration of T cells into the kidneys of AngII mice and AngII‐salt rats are not clear and currently under investigation. Moreover, since AngII‐salt hypertension was not associated with increased T‐cell infiltration, it is perhaps unsurprising that neither RDNX nor renal‐CAP effected T‐cell recruitment to the kidneys.

Although RDNX had no effect on the AngII‐salt model of hypertension in this study, several studies suggest this model is neurogenic and driven by sympathetic activity to the splanchnic vascular bed rather than the kidneys (King et al. 2007; Yoshimoto et al. 2010; Kuroki et al. 2012). AngII‐salt hypertension is attenuated by intracerebroventricular administration of the sodium channel blocker benzamil (Osborn et al. 2014), and celiac ganglionectomy attenuates the development of hypertension in this model (King et al. 2007). Although we have not established the precise mechanism by which celiac ganglionectomy mitigates hypertension in this model, it is interesting to note that a recent study found that denervation of the spleen, which receives its innervation via the celiac ganglion, prevented AngII‐induced hypertension in the mouse and this was hypothesized to be due to attenuation of the immune response to AngII (Carnevale et al. 2016). This suggests that the contribution of a neurogenic immune response to AngII‐induced hypertension may involve not only the kidney but other organs as well.

### Perspectives

The vast discrepancies between models regarding the effects of RDNX on hypertension demonstrate the complex and diverse pathogenic processes that chronically increase arterial pressure. Clearly, much work is still needed to determine under which conditions patients would benefit from RDNX and whether selective ablation procedures might be appropriate. For instance, simple methods to determine whether efferent or afferent renal nerve activity is elevated would be extremely helpful. Measuring neurotransmitters in the urine may be one approach. In addition, because the efficacy of RDNX appears to be associated with renal inflammation, inflammatory markers may also be useful indicators. Our current work is focused on identifying biomarkers that are associated with elevated nerve activity and inflammation in order to better target this treatment to certain forms of hypertension.

## Conflict of interest

None declared.
